# Effect of short-term exposure to ambient air particulate matter on incidence of delirium in a surgical population

**DOI:** 10.1038/s41598-017-15280-1

**Published:** 2017-11-13

**Authors:** Lu Che, Yan Li, Cheng Gan

**Affiliations:** 1 Ninth Department of Plastic Surgery, Plastic Surgery Hospital, Chinese Academy of Medical Sciences, 100144 Beijing, China; 2 Department of Anesthesiology, Peking Union Medical College Hospital, Beijing, 100073 China; 3 National Healthcare Data Center, Affiliated to National Center for Medical Service Administration, 100191 Beijing, China; 4 Hospital Administration Department, Peking University, No. 38 Xueyuan Road, Beijing, 100191 China

## Abstract

Delirium remains an independent risk factor for morbidity and mortality among older surgical adults. Recent research has shed light on the relationship between pollution and dementia, yet little is known about the health impacts of particulate matter (PM) on delirium. Therefore, we aim to further explore association of PM and delirium among surgical population. We conducted a time-stratified case-crossover study. Electronic hospitalization summary reports derived from 26 major cities in China between 1 January 2014 and 31 December 2015 were used. Conditional logistic regression were applied to explore the association between perioperative PM exposure and delirium. A total of 559 surgical patients with delirium were identified. Both PM2.5 and SO_2_ on the day of surgery had a negative impact, with an interquartile range (IQR) increase in PM2.5 (47.5 μg/*m* ^3^) and SO_2_ (22.2 μg/*m* ^3^) significantly associated with an 8.79% (95% confidence interval [CI], 0.01–18.47%, P < 0.05) and 16.83% (95% CI, 0.10–36.35%, P < 0.05) increase in incidence of delirium, respectively. PM on other days during the perioperative period showed no significant impact. The present study showed that short-term exposure to ambient air PM on the day of surgery increased the incidence of delirium in a surgical population during hospitalization.

## Introduction

Delirium is defined as a disorder of global cerebral dysfunction characterized by disordered awareness, attention, and cognition. It is a common syndrome in hospitalized adults, especially elderly populations, with an estimated occurrence of up to 50%^1^. Delirium has been proved to be associated with poor outcomes including functional decline, longer hospitalization, greater health care costs, and higher mortality^2^. Furthermore, the acute onset of altered mental status, hallmarked by difficulty in sustaining attention and a fluctuating course, frequently causes distress among patients and families^3^. In surgical settings, the elderly population is at increased risk for postoperative delirium. Although the etiology of delirium remains elusive, several risk factors have been proposed, including pre-existing dementia, older age, functional impairment, greater comorbidities, and psychopathological symptoms.

According to the Global Burden of Disease Study, air pollution is considered to be the 12th leading global risk factor for reduced disability-adjusted life years^4^, with 3.3 million annual premature deaths reported in 2013. As a developing country with rapid industrialization, China is facing a major environmental crisis^5^. The serious air pollution and possibly massive adverse health impacts has attracted extensive public concern^6^. Whereas the cardiorespiratory effects of exposure to ambient air pollution have been well documented, neurological effects are only recently becoming widely recognized. There is accumulating evidence indicating a potential impact of ambient air pollution on stroke^7^ and cognitive function impairment in older adults^8,9^. However, little information exists regarding the association between pollution exposure and delirium. Thus, the objective of this study was to examine the short-term effects of ambient particulate matter (PM) on incidence of delirium among a surgical population in China.

## Methods

### Study population

The data on incidence of delirium among the surgical population in the present study were sourced from electronic hospitalization summary reports (HSRs) of the top-ranked hospitals (Grade 3 A) according to the National Hospital Performance Evaluation Project of the National Healthcare Data Center of China^10^. This database is derived from 26 major cities in China and includes four municipalities, 21 of 28 provincial capital cities, and Dalian City. Locations of the 26 Chinese cities in this study. Arabic numeral for each city indicates the average daily PM2.5 concentration over the study period (2014–2015) has already been published^11^.(Supplemental material).

The medical information recorded on the HSR includes basic demographics (sex and age), dates of admission, surgical intervention and discharge, hospitalization and discharge diagnoses and their corresponding International Classification of Diseases, 10th Revision (ICD-10) codes, treatments (mainly surgical information), discharge status (survival status, drug allergy, and infection during hospitalization), and financial costs. We identified in-hospital delirium (ICD-10 code F05) among the surgical population from 1 January 2014 to 31 December 2015. We then further specified the timing of delirium diagnosis between the beginning of the surgical procedure and the patient’s death or discharge from the hospital to ensure we enrolled surgical patients with postoperative delirium.

### Data of air pollution

Air pollution is a complex mixture of PM, gases, and organic and inorganic compounds. Data on air pollution, including levels of PM2.5 (particulate matter with aerodynamic diameter <2.5 μm), PM10 (particulate matter with aerodynamic diameter >2.5 to <10 μm), sulfur dioxide (SO_2_), nitrogen dioxide (NO_2_), and carbon monoxide (CO) between 1 January 2014 and 31 December 2015 were obtained from the National Air Pollution Monitoring System. There are 4–15 ambient air monitoring stations in each city. Each monitoring station provides hourly air pollution data to the China National Air Pollution Monitoring System, which is mandated by the government. For each city, we obtained daily (24-hour) mean concentrations for pollutants, averaged across air pollution monitoring stations^12^. To allow adjustment for weather conditions, meteorological data of daily 24-hour average temperature (°C) and relative humidity (%) monitored at meteorological observatories in each city were obtained from the Chinese Meteorological Bureau.

### Study design

We performed pooled analyses, for which observations for all cities were combined. Each city has a special indicator in the dataset. Associations between ambient PM concentrations and delirium were investigated using a time-stratified case-crossover study design. In this design, cases serve as their own controls by using exposure on the days before and after the surgical procedure^13^. For each case of delirium, ambient PM exposure on the day of surgery was compared with exposure on a series of referent days occurring on the same days of the week within the same month and year. This approach can control for the influence of day of the week, seasonal and long-term trends, and slowly varying individual-level risk factors.

### Ethical approval

The present study is considered exempt since the data used was collected for administrative purpose without personal identifiers.

### Statistical analysis

Spearman’s correlation tests were used to examine associations among the exposure variables. Conditional logistic regression was applied to estimate the associations between PM and delirium. To adjust for the delayed and nonlinear effects of temperature and humidity, we used distributed lag non-linear models with three degrees of freedom in the natural cubic splines and a maximum lag of 3 days^14^. To control for spatial variations in the health effects of meteorological conditions, interactions between the meteorological variables and cities were also included in the model. We also incorporated public holidays in the model. The results are reported as the percentage change and 95% confidence interval (CI) in the relative risk of delirium per interquartile range (IQR) increase in PM concentration.

To examine the temporal association of PM concentration with delirium, we examined the impact of pollution in a perioperative manner, collecting data from 3 days before surgery to the day of surgery. We fitted the models with different lag structures from the day of operation (lag0) up to 3 lag days (lag3).

Stratified analyses were used to examine whether associations differed by sex, age (≥65 years and <65 years) and comorbidities including diabetes mellitus (DM), hypertension, ischemic heart disease (IHD), chronic obstructive pulmonary disease (COPD), and Alzheimer disease (AD). Stratified models were compared using a Z-test^15^.

All analyses were conducted using R programming language (V.3.2.2, R Development Core Team). All statistical tests were two-sided, and P < 0.05 was considered statistically significant.

## Results

### Demographic and pollution variables

We identified a total of 559 surgical patients with delirium that was newly diagnosed during hospitalization (Table 1). The mean age of this group was 60.4 years, and 66.9% of patients were men.

**Table 1:** Demographic characteristics of hospital induced delirium.

Variable	Patients with surgery
Total	559
Gender	
Male (%)	374 (66.9)
Female (%)	185 (33.1)
Age (year) (mean ± SD)	60.4 ± 20.9
<65 (%)	295 (52.8)
≥65 (%)	264 (47.2)
Hypertension (%)	165 (29.5)
Diabetes (%)	107 (19.1)
IHD (%)	78 (14.0)
COPD (%)	41 (7.3)
AD (%)	17 (3.0)

Summary statistics of air pollutants and meteorological variables during the study period in the 26 Chinese cities investigated are presented in Table 2. The means (SD) of air pollutants were 63.5 (50.6) μg/*m* ^3^ for PM2.5, 106.8 (71.9) μg/*m* ^3^ for PM10, 29.6 (32.6) μg/*m* ^3^ for SO_2_, 44.1 (19.4) μg/*m* ^3^ for NO_2_, and 1.15 (0.63) μg/*m* ^3^ for CO. Means (SD) of temperature and relative humidity were 14.5 °C (10.9 °C) and 69.2% (33.2%), respectively.

**Table 2:** Summary statistics for air pollutant concentrations and meteorological variables.

Variable	Mean ± SD	Minimum	Percentile			Maximum	IQR
			25th	50th	75th		
PM2.5 (μg/*m* ^3^)	63.5 ± 50.6	5.1	31.5	49.4	79.0	897.5	47.5
PM10 (μg/*m* ^3^)	106.8 ± 71.9	7.4	58.3	89.4	135.2	977.3	76.9
SO_2_ (μg/*m* ^3^)	29.6 ± 32.6	1.9	11.4	18.8	33.6	316.9	22.2
NO_2_ (μg/*m* ^3^)	44.1 ± 19.4	4.5	30.0	40.2	54.1	175.8	24.1
CO (μg/*m* ^3^)	1.15 ± 0.63	0.14	0.76	0.99	1.32	8.41	0.56
Temperature (°C)	14.5 ± 10.9	−25.7	7.0	16.4	23.3	35.5	16.3
Relative humidity (%)	69.2 ± 33.2	8	53	69	80	97	27

### Associations between air pollution and delirium

**Figure 1:**
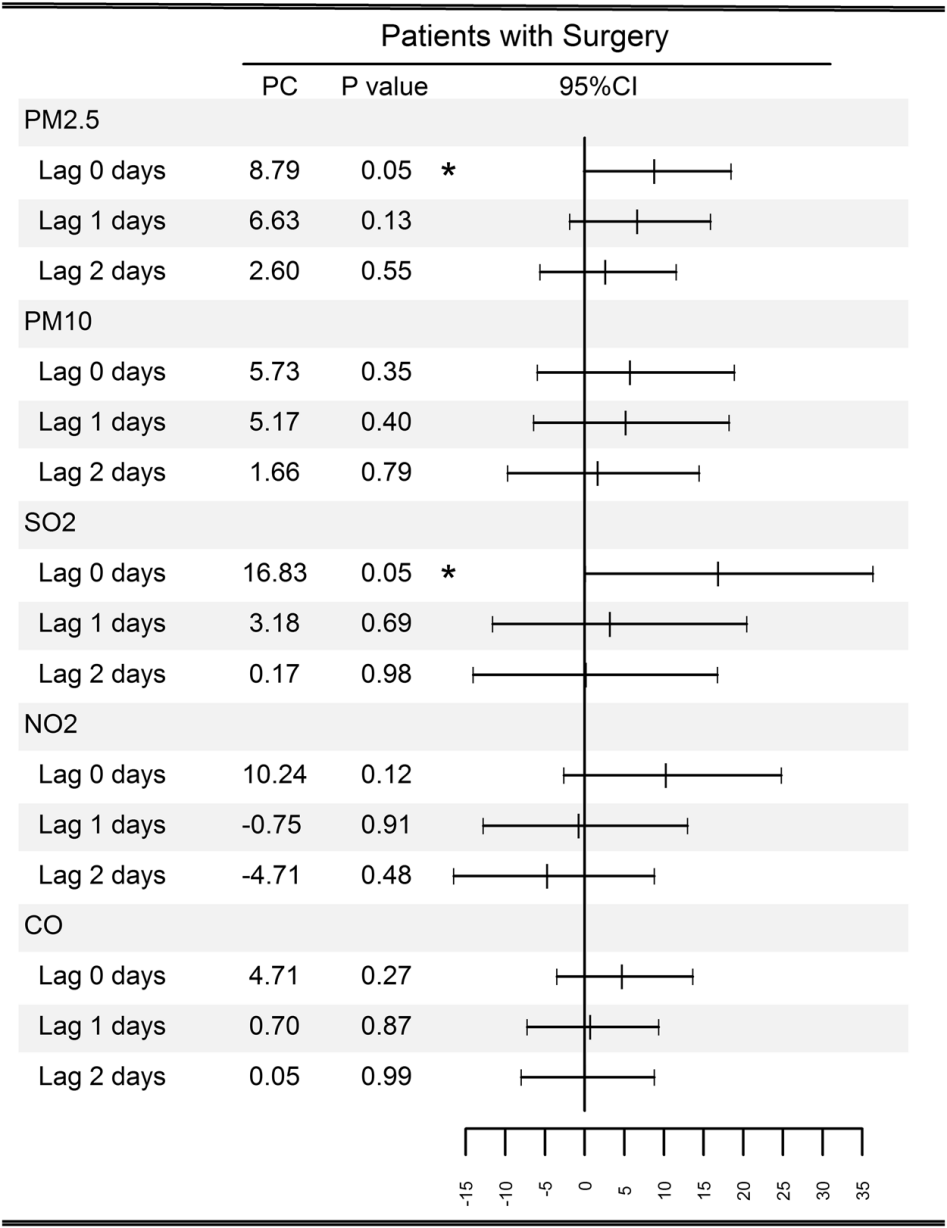
Percentage change (PC) with 95% confidence interval (CI) among surgical patients with delirium associated with an interquartile range increase in air pollutant concentration for different lags, in 26 Chinese cities during 2014–2015. ˚*P* < 0.1, *P < 0.05.

There were clear exposure–response associations between average PM2.5 and SO_2_ concentrations on the day of surgery and delirium during hospitalization, for all 26 cities (Fig. 1). Percentage changes with 95% CIs in occurrence of delirium among surgical patients associated with an IQR increase in PM2.5 (47.5 μg/*m* ^3^) and SO_2_ (22.2 μg/*m* ^3^) concentrations for perioperative days are displayed in Fig. 1. An IQR increase in PM2.5 and SO_2_ concentrations on the day of surgery corresponded to an 8.79% (95% CI, 0.01–18.47%) and 16.83% (95% CI, 0.1–36.35%) increase in delirium, respectively. We found no evidence of a significant negative impact of PM10, NO_2_, or CO on surgical patients for risk of delirium at all the examined time points (all P > 0.05) (Fig. 1).

**Figure 2:**
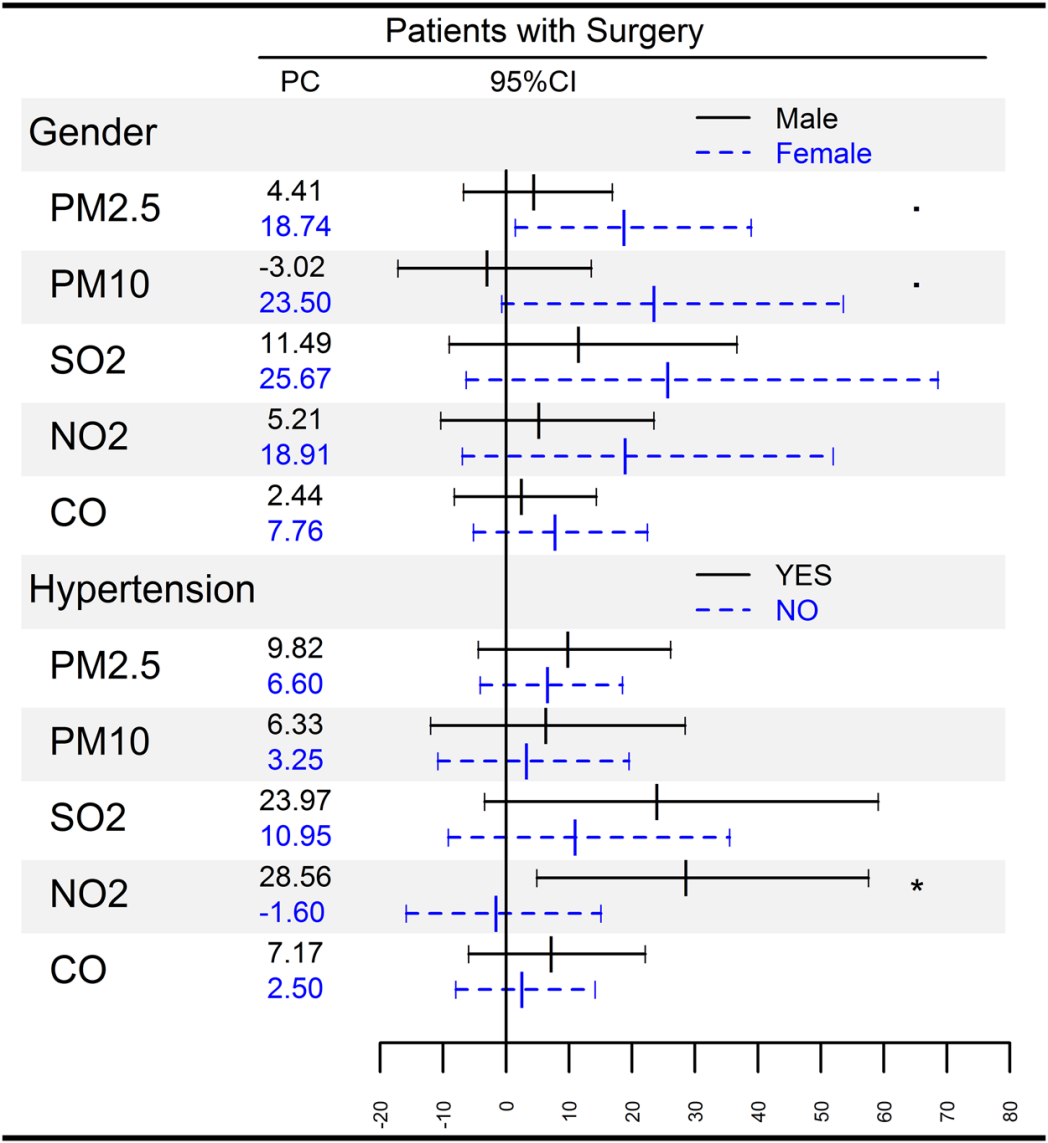
Percentage change (PC) with 95% confidence interval (CI) among surgical patients with delirium with an interquartile range increase in PM2.5 (47.5 μg/*m* ^3^), PM10 (76.9 μg/*m* ^3^), SO_2_ (22.2 μg/*m* ^3^), NO_2_ (2.41 μg/*m* ^3^), CO (0.56 μg/*m* ^3^) concentrations, stratified by sex and hypertension at lag 0 days. ˚P < 0.1, *P < 0.05.

Figure 2 shows the associations between PM concentration on the day of surgery and delirium stratified by sex and hypertension. The impact from PM2.5 was stronger among female patients, with an 18.74% (95% CI, 1.49–38.92%.) increase of delirium with an IQR increase of PM2.5. Also, among patients with hypertension, an IQR increase in NO_2_ concentrations on the day of surgery was associated with a 28.56% (95% CI, 4.88–57.58%) increase in risk of developing delirium during hospitalization.

We found no evidence for effect modification by age or other comorbidities, including IHD, COPD, DM, and AD.

## Discussion

Delirium in elderly surgical patients is of great concern among heath care providers. However, it remains poorly understood owing to both the heterogeneous nature of delirium and the methods used to research it^16^.

In this study, we report results from 26 large Chinese cities concerning the short-term effects of ambient PM pollution on delirium during hospitalization among surgical patients. To the best of our knowledge, this is the first epidemiologic study worldwide to date to examine the association of PM with delirium. Here, we report a possible link between short-term pollution exposure and newly diagnosed delirium during hospitalization using a time-stratified case-crossover study design.

The impact of pollution on cardiovascular disease has been widely researched and underlying systemic inflammation has been suggested as a possible mechanism; however, *in vivo* evidence is still lacking. Short-term and long-term exposures to outdoor PM pollution are associated with a range of adverse cardiovascular health effects such as atherosclerosis, myocardial ischemia, myocardial infarction, stroke, and death. Similar observations have been made for a series of neurological conditions including headache (Loane *et al*., 2013), depression (Wellenius *et al*., 2015), autism (Suades-Gonzalez *et al*., 2015), neurodegenerative disease^17^, and cognitive function decline^8,9,18^.

Pathways through which air pollution may impact cognitive function are poorly understood. Components of air pollution can produce proinflammatory responses, thereby instigating a systemic-induced cytokine response originating in peripheral organs, and these components may eventually be transferred to the brain. It has been suggested that PM, especially of small diameter, may have the potential to extend beyond pulmonary organs to the central nervous system (CNS)^19^. Furthermore, toxic substances may penetrate the brain via the olfactory epithelium; olfactory dysfunction is among the earliest features of AD and Parkinson disease^17^. The resulting neuroinflammation and brain oxidative stress exerted by short-term exposure to pollution may be the cause of increased risk of CNS deficit, such as delirium.

The fact that delirium has an acute presentation and is difficult to diagnose make it a challenge to study; therefore, few studies have explored the possible link between pollution and delirium. A growing body of research has highlighted the negative impact of pollution on cognitive function among older adults^20^. It is believed that dementia and delirium share some common pathways. Research has showed that delirium is an independent predictor of sustained poor cognitive and functional status whereas dementia is a known risk factor for delirium^2^. It has been suggested that inflammatory processes appear to play an important role in the pathogenesis and clinical course of cognitive impairment in delirium^21^.

Another possible link between heavy pollution and delirium among surgical patients is through disruption of circadian rhythms, particularly owing to inadequate daytime illumination. It has been well established that maintaining healthy circadian periodicity can help to prevent delirium^22^. Early morning bright light has been found to enhance sleep, lower the prevalence of arrhythmia, and lower rates of delirium. Inadequate amounts of daylight owing to high pollution levels can serve as an underlying contributing factor of delirium. It has been shown that short-term exposure to air pollution can lead to disturbance in the circadian rhythms of renal sodium handling and blood pressure^23^, suggesting a more profound impact of pollution on circadian rhythms apart from lighting. Sufficient lighting during the day and melatonergic interventions during periods of heavy pollution warrant testing in future clinical trials.

It has also been noted that depression and mood disorders have been associated with air pollution. PM10, PM2.5, NO_2_, CO, SO_2_ are all associated with depression and emotional symptoms in the elderly. Dementia, delirium, and depression syndromes have a high degree of overlap, can exist simultaneously in the same patient, and often increase the risk for each other^24^. Recent literature has suggested that the impacts of PM2.5 on cognition are mediated by mood disorders such as depression^25^. The possible impact of short-term pollution exposure on mood in surgical patients should also be considered in future research.

Previous studies attempting to elucidate whether sex is a risk factor for delirium have yielded mixed results^26^. In this study, we noticed that women were more prone to be influenced by pollution in terms of delirium risk. This susceptibility to ambient pollution by sex has been noted previously. It has been reported that exposure to PM2.5 has a significant association with carotid artery intima thickening^27^, and this impact is more pronounced among women. Women are thought to have less cognitive functional capacity and experience greater levels of disability or frailty than men^28^. Elevated levels of anxiety may be one reason underlying why women are more susceptible than men to the effects of ambient pollution on delirium^25^.

### Limitations of this study

Owing to limited data, the hypothetical role of some risk factors such as advanced age, previous diagnosis of dementia, and specific comorbidities or operative risk factors such as operation duration remain to be clarified. In addition, in this study, we did not fit two-pollutant models to examine the robustness of associations between PM and delirium because collinearity among the pollutants added uncertainty to interpretation of the results and limited our ability to isolate the independent effects of PM on delirium.

## Conclusion

We found that short-term exposure to high levels of PM2.5 and SO_2_ are significantly associated with increased risk of delirium in a surgical population in 26 major cities of China. We also observed effect modification by sex, with significantly stronger associations in female surgical patients. Therefore, we suggest that ambient PM may be a potential risk factor for delirium. Clinicians should actively implement prevention strategies prior to surgery. Further research is required to confirm our findings and shed light on the mechanisms connecting these disorders.

## Electronic supplementary material

Supplementary information
