# Supplementary material for: Effect of short-term exposure to ambient air particulate matter on incidence of delirium in a surgical population
Source: Sci Rep. 2017 Nov 13;7:15461. doi: 10.1038/s41598-017-15280-1 (PMC5684401; doi:10.1038/s41598-017-15280-1)
Supplement: Supplementary file 1 — Supplementary information [file 41598_2017_15280_MOESM1_ESM.doc]

Effect of short-term exposure to ambient air particulate matter on incidence of delirium in a surgical population

Lu Che ^1^, Yan Li^2,3^, Cheng Gan^4^

1. Department of Anesthesiology, Peking Union Medical College Hospital, 100073, Beijing, China

2. Hospital Administration Department, Peking University, No.38 Xueyuan Road, Beijing 100191, China

3. National Healthcare Data Center, Affiliated to National Center for Medical Service Administration, 100191 Beijing, China

4. Ninth Department of Plastic Surgery, Plastic Surgery Hospital, Chinese Academy of Medical Sciences, Badachu Road 33#, Beijing 100144, China

Corresponding author: Cheng Gan, MD

E-mail: gnche@sina.com

Address: Ninth Department of Plastic Surgery, Plastic Surgery Hospital, Chinese Academy of Medical Sciences, Badachu Road 33#, Beijing 100144, China

Telephone: 15901045120

Running head: Effect of pollution on delirium

Supplemental material

Table 1. Spearman correlation coefficients among the exposure variables.

| Variables | PM2.5 | PM10 | NO2 | SO2 | CO | Temp | RH |
| --- | --- | --- | --- | --- | --- | --- | --- |
| PM2.5 | 1.00 | 0.87* | 0.67* | 0.61* | 0.68* | -0.32* | -0.08* |
| PM10 | ― | 1.00* | 0.64* | 0.63* | 0.60* | -0.27* | -0.33* |
| NO_2_ | ― | ― | 1.00 | 0.54* | 0.59* | -0.36* | -0.11* |
| SO_2_ | ― | ― | ― | 1.00 | 0.55* | -0.51* | -0.36* |
| CO | ― | ― | ― | ― | 1.00 | -0.37* | 0.02† |
| Temp | ― | ― | ― | ― | ― | 1.00 | 0.18* |
| RH | ― | ― | ― | ― | ― | ― | 1.00 |

*P < 0.001,†P < 0.05.
